# Short-term assessment of BCR repertoires of SLE patients after high dose glucocorticoid therapy with high-throughput sequencing

**DOI:** 10.1186/s40064-016-1709-4

**Published:** 2016-01-26

**Authors:** Bin Shi, Jiang Yu, Long Ma, Qingqing Ma, Chunmei Liu, Suhong Sun, Rui Ma, Xinsheng Yao

**Affiliations:** Department of Immunology, Research Center for Medicine and Biology, Innovation and Practice Base for Graduate Students Education, Zunyi Medical University, Zunyi, 563000 China; Department of Laboratory Medicine, Zunyi Medical University, Zunyi, 563000 China; Department of Breast Surgery, The First Affiliated Hospital of ZunYi Medical University, Zunyi, China; Cell Engineering Laboratory, The First Affiliated Hospital of ZunYi Medical University, Zunyi, China; Department of Nephropathy and Rheumatology, The First Affiliated Hospital of ZunYi Medical University, Zunyi, China; Central Laboratory, Guizhou Aerospace Hospital, Zunyi, China

**Keywords:** SLE, BCR repertoire, H-CDR3, High-throughput sequencing

## Abstract

We analyze and assess BCR repertoires of SLE patients before and after high dose glucocorticoid therapy to address two fundamental questions: (1) After the treatment, how the BCR repertoire of SLE patient change on the clone level? (2) How to screen putative autoantibody clone set from BCR repertoire of SLE patients? The PBMCs of two SLE patients (P1 and P2) at different time points were collected, and DNA of these samples were extracted. High-throughput sequencing technology was applied in detection of BCR repertoire. Finally, we used bioinformatic methodology to analyse sequence data. We found that these two patients lost some IGHV3 family genes usage after treatment compared with before treatment. For pairing of IGHV–IGHJ gene, no significant change was shown for each patient. In addition, analyses of the composition of H-CDR3 showed overall AA compositions of H-CDR3 at three time points in each SLE patients were very similar, and the results of H-CDR3 AA usage that had the same length (14 AA) and the same position were similar. Antinuclear antibody tests of SLE patients showed that level of some antinuclear antibodies reduced after treatment; however, there was no sign that the percentage of autoantibody clones in BCR repertoires would reduce. High dose glucocorticoid treatment in short term will have little impact on composition of BCR repertoire of SLE patient. Treatment can reduce the amount of autoantibody in the protein level, but may not reduce the percentage of autoantibody clones in BCR repertoire in the clonal level.

**Electronic supplementary material:**

The online version of this article (doi:10.1186/s40064-016-1709-4) contains supplementary material, which is available to authorized users.

## Background

Systemic lupus erythematosus (SLE) is an autoimmune disease with unknown etiology and abnormal activation of B cells. Various autoantibodies can be detected in the serum of the SLE patients. Among these autoantibodies, anti-dsDNA, anti-SM and anticardiolipin antibodies have important diagnosis value (Hochberg 1997). It is currently considered that, autoreactive B cell and the autoantibodies secreted by plasmocyte are the main factors that directly resulted in pathogen of SLE (Arbuckle et al. 2003). Meanwhile, B cell is also considered as the main target of SLE treatment (Shlomchik et al. 2001; Sanz and Lee 2010).

B cell receptor (BCR), which is on the surface of B cell membrane, is an important functional receptor of B cell, involving in immune response of humoral inducing. BCR is a tetrapeptide chain structure with two heavy chains (IGH) and two light chains (IGL). The heavy chain complementary determining region 3 (H-CDR3) is thought to be the key regions of antigen recognition and combination (Tonegawa 1983; Chothia et al. 1989; Padlan 1994; Wilson and Stanfield 1994). As for healthy people, peripheral blood often contains about 3 × 10^9^ BCRs, and the diversity of BCR repertoire or antibody repertoire is produced by multiple mechanism, mainly including rearrangement of various discontinuous V, D and J gene segments (recombination diversity) (Jung et al. 2006), insertion and deletion of nucleotide at VDJ joint (junctional diversity) (Stewart and Schwartz 1994) and somatic hypermutation (SHM) after B cell entering peripheral region (Berek et al. 1991).

The past studies have done distinctive analysis on BCR gene composition and rearrangement of SLE and functional study on SLE autoantibody. Kasaian et al. (1994) found that many VH and VL genes taken from anti-DNA IgA autoantibody heavy chain can improve the choice of its SHM. Mockridge et al. (1998) has analyzed on recombination of VH3-34 and VL gene of two SLE patients’ autoantibody and provided a good basis for studying the length and specificity of CDR3 amino acid (AA). In 1996, Krishnan et al. found that SLE anti-dsDNA autoantibody was closely related to content of arginine of H-CDR3 (Krishnan et al. 1996), and not long after that, found that there was no significant difference in arginine usage of H-CDR3 region in anti-DNA autoantibody between NZBxNZW F1 mice and BALB/c mice in the early stage. However, oligoclonal hyperplasia will gradually occur in H-CDR3 of autoantibody rich in arginine in NZBxNZW F1 mice (Krishnan and Marion 1998). Guo et al. who studied on SLE mouse model found that high affinity antinuclear antibody mainly come from gene recombination, SHM and VH gene replacement of CDR3 region, and that the SHM detection of autoantibody CDR3 region was very important in the study of SLE autoantibody development and B cell differentiation and could provide good monitoring points for SLE (Guo et al. 2010). Although anti-dsDNA and anti-APL are very important in SLE pathology, it is not mean that if there is anti-dsDNA and anti-APL, there will be clinical manifestation. Only there is arginine gathering in IgG CDR3 region, there will be serious pathology lesion (Rahman 2004).

On clinical, high dose glucocorticoid therapy has been widely used in SLE treatment at the active stage. However, this treatment is nonspecific, unable to distinguish normal cells and autoreactive cells, so as to cause large amount of side effects, including serious toxicity and damage to metabolic balance, cardiovascular system, eyes, and bone. Currently, it is still unclear whether this non-specific treatment will affect BCR repertoire of SLE patients. In order to overcome these side effects, some scholars had researched and developed B cell targeted drugs (some monoclonal antibodies aimed at B cell membrane molecules or excreted factors). Unfortunately, B cell targeted therapy strategy cannot act specifically on autoreactive B cells. In addition, the type of SLE autoantibodies is various; therefore, we recommend the clone set of autoantibodies is the better target of targeted therapy.

On the consideration of the listed reasons, we used high-throughput sequencing to make a short-term evaluation on BCR repertoire of SLE patients after high dose glucocorticoid treatment, aiming at solving two fundamental problems: (1) After treatment, how the BCR repertoire of SLE patient change on the clonal level? (2) How to screen putative autoantibody clone set from SLE BCR repertoire? Because there were CNV (copy number variation) and germline gene variation on the IGHV gene locus, there is great difference in IGHVs between individuals (Watson and Breden 2012). Besides, SLE patients have great heterogeneity in clinical and serology manifestation. On clinical, it is suggested to conduct personalized management and treatment on SLE patients. Based on these reasons, the experiment we designed is to observe the change of same SLE patient before and after treatment. Generally, the renewal cycle of human immune cell is 28 days, so we chose the first month after treatment as the first time point after treatment. On the basis of this, we further track the prognosis condition of the patients at the third month after treatment.

## Methods

### Subjects

Following the principle of informed consent and under a protocol approved by local Ethics committee, we collected the peripheral blood samples of 2 SLE patients that are provided by Department of Nephropathy and Rheumatology of Zunyi Medical University affiliated hospital. SLE patients were diagnosed according to the standard proposed by American College of Rheumatology in 2011 and those during gestation or lactation period and those with infections or serious primary disease of heart, liver, renal, brain and hemopoietic system or other autoimmune disease were eliminated. The result codes of three time points, before treatment, 1 month after treatment and 3 months after treatment, of P1 (female, 45 years old, recurrent lupus nephritis) were defined as P1-0, P1-1 and P1-3. Similarly, P2-0, P2-1 and P2-3 represented the result of these three time points of patient P2 (male, 20 years old, primary lupus nephritis), respectively. The basic information of patients was presented in Additional file 2: Table S1. These two patients both received high dose glucocorticoid therapy (≥500 mg).

### Preparation of sample

Ficoll lymphocyte separation medium (Beijing Solarbio Science and Technology Co., Ltd, Cat. No. P8610) and density gradient centrifugation were used in this process. Peripheral blood mononuclear cells (PBMCs) of every patient at different time points were separated. QIAamp DNA MiniKit (Cat. No. 51304, QIAGEN) was used to extract genomic DNA (stored under −20 °C) from 6 peripheral blood samples of 2 patients.

#### Count of peripheral white blood cell and detection of C3 and anti-nuclear antibody spectrum

Peripheral white blood cell count and detection of C3 and anti-nuclear antibody spectrum at every time point were provided by Medical Laboratory of Zunyi Medical University affiliated hospital. The method of peripheral white blood cell count was flow type laser impedance triple method (full automatic blood cell analyzer assembly line, XE-5000, Sysmex). Method of C3 detection was immunoturbidimetry (full automatic special protein analyzer, IMMAGE800, Beckman). The detecting method of antinuclear antibody spectrum was EURO Blot (according to standard operating process of the factory).

### High-throughput sequencing

Before high-throughput sequencing, the concentration and purity of DNA of samples need to reach the requirement of BCR CDR3 sequencing and the volume of every sample was the same (the whole total DNA needs to reach 2 μg). Multiplexed PCR amplification is performed to amplify rearranged CDR3 sequences, designing an upstream primer and downstream primer in the VH functional gene region and JH functional gene region, respectively. Every primer was set in the specific site of BCR H chain. Error from bias in this multiplexed PCR assay was controlled using synthetic templates (Carlson et al. 2013). Illumina adaptors are subsequently added for next-generation sequencing. Amplification and sequencing were completed by Adaptive Biotechnologies ImmunoSEQ platform (http://www.adaptivebiotech.com) (Wu et al. 2014).

### Sequence analysis

Raw sequences with FASTA format were submitted to IMGT/HighV-QUEST online software (http://www.imgt.org). IMGT summary file was used to filter sequence according to the following principle: (1) No results; (2) Unknown; (3) Warnings; (4) Unproductive; (5) V gene was pseudogene; (6) AA of 104 position was non-cysteine (Cysteine, C) or AA of 118 position was non-tryptophan (Tryptophan, W). The analysis and storage of sequences were completed in Excel (version 2010) and graph was completed by Prism 5 software (GraphPad). The formula of inverse Simpson index (1/Ds) was 1/Ds = 1/[1-∑(Ni(Ni-1))/(N(N-1))], Ni was defined as the frequency of i gene, and N was defined as the total number of genes.

## Results

### Clinical hematology examination

One month after high dose glucocorticoid therapy, the number of white blood cell of two SLE patients both decreased, and on the third month, the white blood cell number rose to the level before treatment (Additional file 2: Table S2). After treatment, the clinical manifestation of two patients turned better, but serumal IgG level of P2 decreased evidently while serumal IgG level of P1 increased. In addition, the serumal antinuclear antibody level of two SLE patients had different degree of change at three different time points, which reflected serological heterogeneity of SLE (Table 1). The antinuclear antibody level of two patients both improved, indicating that the treatment can partly decrease the expression of autoantibody.

**Table 1:** The antinuclear antibodies indexes of two SLE patients at different time points

Antinuclear antibodies	P1	P2
Anti-nRNP/SM	3+, 2+, 2+	3+, 2+, 1+
Anti-SM	N, N, N	2+, 1+, N
Anti-SSA	3+, 3+, 3+	3+, 3+, 3+
Anti-RO-52	N, N, N	3+, 3+, 3+
Anti-SSB	N, N, N	1+, N, N
Anti-SCOL-70	N, N, N	N, N, N
Anti-JO-1	N, N, N	N, N, N
Anti-CENP B	N, N, N	N, N, N
Anti-dsDNA	3+, 2+, 2+	1+, N, N
Anti-nucleosome	3+, 3+, 3+	N, N, N
Anti-histone	3+, 1+, 1+	N, N, N
Anti-ribosomal P	N, N, N	3+, 3+, 2+
ACA	N, N, N	N, N, N

The “N” represents negative result, the “1+” represents weakly positive result, “2+” represents positive result, “3+” represents strong positive result

### Diversity evaluation of BCR repertoire

After the raw data was uploaded to IMGT/HighV-QUEST, IMGT data was used for analysis after filtration (Table 2). The Table 2 shows that the BCR sequences from patient P1 decreased evidently after treatment while there was no evident change in patient P2. In order to evaluate the change of diversity of BCR repertoire, we introduced inverse Simpson’s diversity index (1/Ds) (Hochberg et al. 2001). The clone distributions of these two SLE patients at three different time points show that the initial diversity of P1 before treatment was evidently lower than that of P2. The diversity of BCR repertoire of P1 after treatment decreased significantly and the clone distribution changed evidently, while the diversity of BCR repertoire of P2 had no evident change over time. These results suggested that the humoral immunity of P1 had steady maladjustment, existing the risk of infection or suffering from other diseases; while the state of humoral immunity of P2 tended to be stable (Additional file 1: Figure S1).

**Table 2:** The sequence data of two SLE patients at different time points

Patients no.	Raw sequences	Analyzed sequences	Analyzed unique H-CDR3
P1-0	1105330 (27206)	493579 (14212)	13949
P1-1	579487 (12468)	255209 (6342)	6180
P1-3	366474 (7574)	151170 (3927)	3865
P2-0	1079045 (53222)	470328 (28027)	27729
P2-1	1176658 (53203)	526024 (28160)	27888
P2-3	1037150 (40748)	465958 (21349)	21077

Data in brackets is unique

### Usage of IGHV gene subgroup

**Fig. 1:**
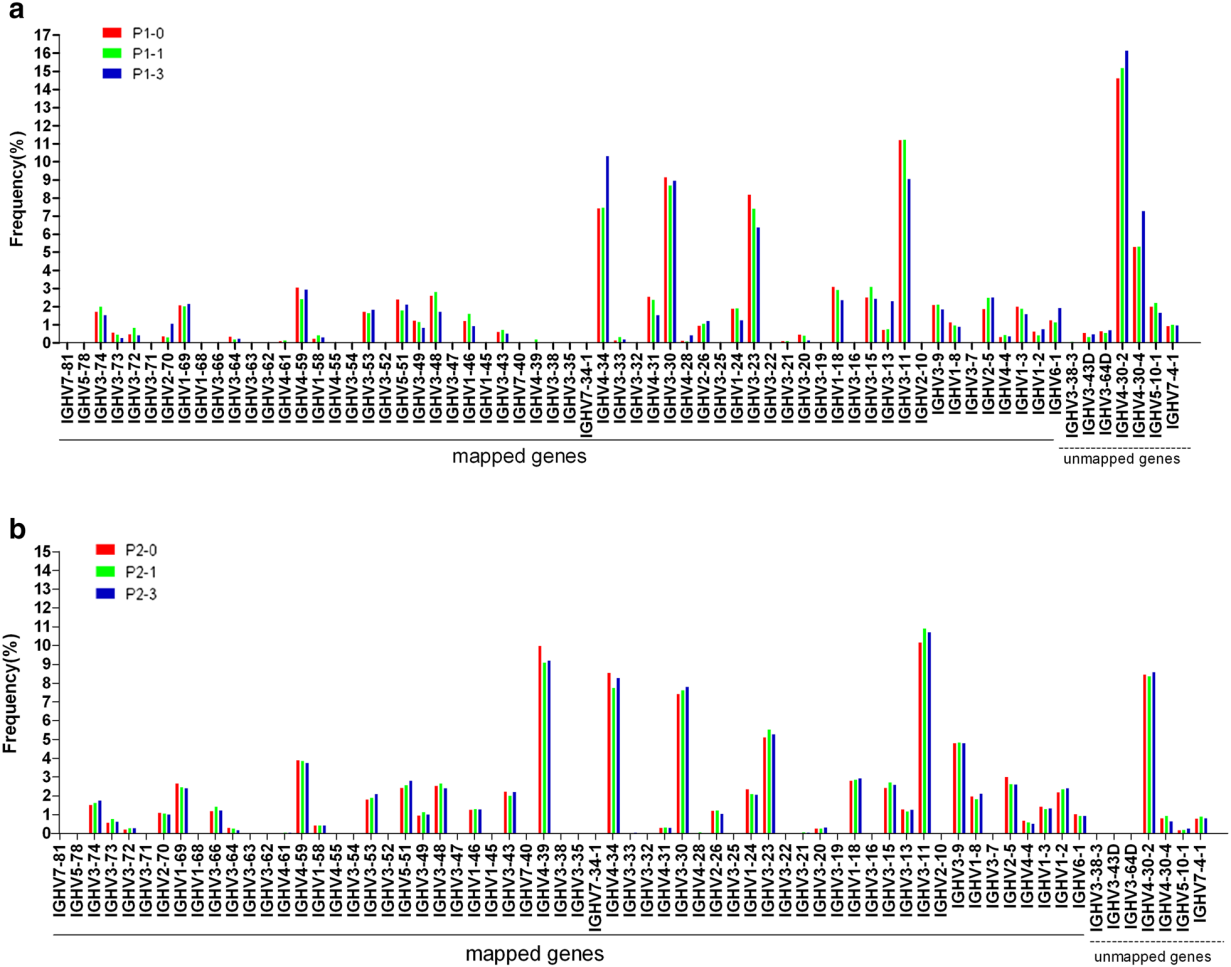
Known alleles for each of the mapped and unmapped IGHV genes are arranged according to IMGT/HighV-QUEST Statistical Analysis Report. And IGHV genes usage of two SLE patients is calculated as the percentage of the total analyzed sequences. **a** P1-0, P1-1 and P1-3; **b** P2-0, P2-1 and P2-3

To evaluate the change of IGHV gene frequency of SLE patients over time, we calculated the frequency of IGHV gene subgroup of two SLE patients at each point (Fig. 1). Figure 1 indicated that IGHV gene distributions of two SLE patients at different time points were similar and the IGHV usage close to C terminal increased evidently. After treatment, only P1 had little change with not over 3 % in some gene usages (IGHV4-34, IGHV3-13, IGHV3-11, and IGHV4-30-4) at the third month after treatment. More notably, after treatment, BCR repertoires of two SLE patients lost some IGHV gene usages. For example, at the first month after treatment, P1 patient lost IGHV3-38, IGHV3-25 and IGHV3-7 usages (Additional file 2: Table S3). At the third month after treatment, some new IGHV genes lost, including IGHV3-35 and IGHV3-38-3. As for P2 patient, at the first month after treatment, no loss of IGHV gene usage was observed. However, at the third month after treatment, usages of IGHV3-35 and IGHV3-7 lost (Additional file 2: Table S3). Surprisingly, at the first month after treatment, P1 got some new IGHV usages with low frequency, including IGHV3-7 (0.0025 %) and IGHV3-64D (0.0086 %). Interestingly, these lost genes were all from IGHV3 family.

### Pairing of IGHV–IGHJ gene

**Fig. 2:**
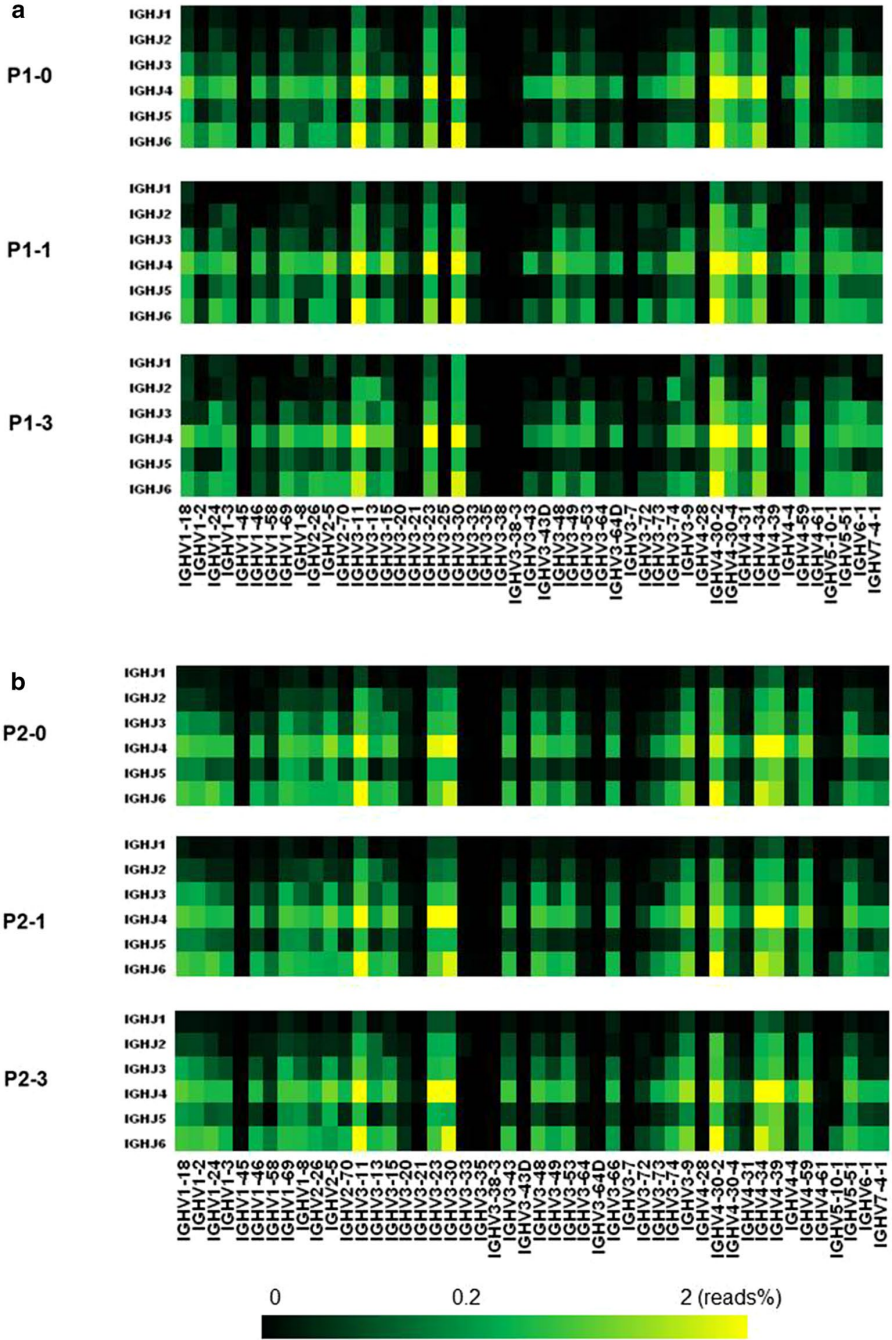
Frequencies of specific IGHV and IGHJ gene pairing in two SLE patients at different time points are depicted. IGHJ genes are indicated on the left, IGHV genes at the bottom of the panels. Total reads (%) of a given pairing are indicated by color code. P1 and P2 show restricted usage of IGHV and IGHJ genes. **a** P1-0, P1-1 and P1-3. **b** P2-0, P2-1 and P2-3

To assess the change of IGHV–IGHJ gene pairing over time, we calculated the frequencies of IGHV–IGHJ pairing of them at three time points (Fig. 2). The heat map in Fig. 2 indicated that IGHV–IGHJ gene pairing of P1 and P2 had no evident change at three time points (Fig. 2). It was worth noting that P1 and P2 had some similar dominant pairing (yellow grid area), namely, IGHV3-11-IGHJ4, IGHV3-11-IGHJ6, IGHV3-23-IGHJ4, IGHV3-30-IGHJ4, IGHV3-30-IGHJ6, IGHV4-30-2-IGHJ4, IGHV4-34-IGHJ4 and IGHV4-30-2-IGHJ6.

### Composition of H-CDR3

**Fig. 3:**
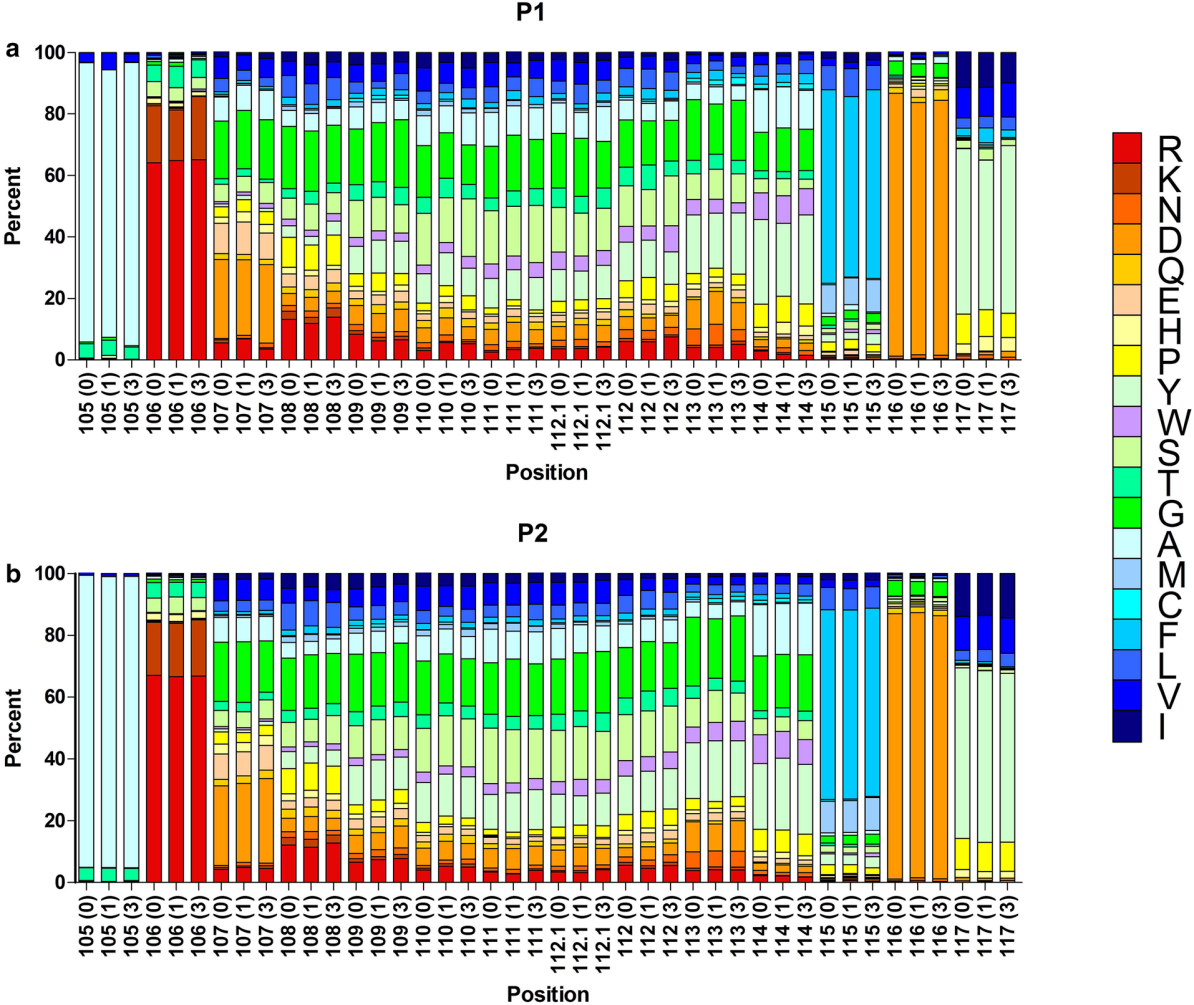
The frequency of individual amino acids at specific positions (105–117) for the most prevalent H-CDR3 sequences having the length of 14 amino acids (the basic length of CDR3-IMGT) in each SLE patient is shown. The color menu for amino acids is according to IMGT (Ruiz and Lefranc 2000). H-CDR3 positions are shown according to the IMGT unique numbering. Arabic numerals (*0, 1, 3*) in the brackets at the bottom of the panels respectively represent three time points. **a** P1-0 (n = 1674), P1-1 (n = 762) and P1-3 (n = 456). **b** P2-0 (n = 3060), P2-1 (n = 3200) and P2-3 (n = 2423)

We analyzed the composition change of unique H-CDR3 over time, including H-CDR3 length distribution and H-CDR3 AA usage. The change of H-CDR3 length distribution of two SLE patients is showed in Additional file 1: Figure S2. H-CDR3 length distribution of P1 was similar between pre-therapy and 1 month after treatment, but was evidently changed at the third month, reflecting that H-CDR3 longer than 15 AA increased (Additional file 1: Figure S2A). It was reported that the longer H-CDR3 may be related to autoimmune response (Ichiyoshi and Casali 1994; Ditzel et al. 1996; Wardemann et al. 2003). As for P2 patient, we observed that H-CDR3 length distributions at different time points nearly had no change (Additional file 1: Figure S2B). The AA usage of unique H-CDR3 of two SLE patients at different time points had no evident change (Additional file 1: Figure S3). Therefore, we further investigated the AA usages of different positions (105–117, according to IMGT unique numbering) of H-CDR3 with the same length. Figure 3 indicated that the AA usages of different positions of the longest H-CDR3 (14 AA) at different time points had no evident change. H-CDR3 AA composition of SLE patients nearly had no change after treatment, indicating that during this period time, these two SLE patients barely had new specific B cell response.

### Screening of autoantibody clone

The occurrence of large amount polyclonal autoantibodies is the key factor that results in pathopoiesis of SLE, therefore, autoantibody clone set should be considered as the commendable treatment target. In order to screen the potential autoantibody clones from BCR repertoire of two SLE patients, we downloaded all the known autoantibody IGH sequences from IMGT/LIGM-DB database, and investigated the sequence number from BCR repertoire of two SLE patients that had the same IGHV, IGHJ and H-CDR3 AA length with the known autoantibody (Table 3; Zhang et al. 2013). Because of the existence of SHM, it is difficult to confirm the difference of H-CDR3 AA. Therefore, the screening condition was not limited to the same H-CDR3 AA sequence. Table 3 indicated that number of potential autoantibody clones of P1 decreased evidently after treatment while number and proportion of potential autoantibody clones of P2 maintained in a relatively steady level. It was worth noting that, although the diversity of BCR repertoire of P1 reduced evidently after treatment, however, the proportion of potential autoantibody clones did not decrease. Considering that autoantibody clones is preferentially selected in SLE, the affinity of clone with lower frequency may be relatively lower. Therefore, we further filtered in the sequences with clone no. ≥50 from these potential autoantibody clones (Additional file 2: Table S4), these sequences were the putative autoantibody clones (Additional file 3: Putative autoantibody clones). Interestingly, the diversity of BCR repertoire of P1 was evidently less than that of P2, but the number of putative autoantibody clones of P1 (recurrence) was evidently more than that of P2 (primary attack).

**Table 3:** Number of heavy chain variable regions that use the same V and J genes and have the same CDR3 length as known autoantibody from IMGT/LIGM-DB

Autoantibody accession no.	IGHVs	IGHJs	CDR3 length	P1-0	P1-1	P1-3	P2-0	P2-1	P2-3
AF035024	IGHV3-30	IGHJ4	6 AA	87	0	0	75	156	36
AF035021	IGHV3-30	IGHJ4	10 AA	1060	375	303	802	896	1353
L12105	IGHV1-2	IGHJ4	10 AA	14	36	38	136	379	227
AF035023	IGHV3-48	IGHJ3	11 AA	65	42	50	58	127	40
L12087	IGHV1-3	IGHJ4	11 AA	416	238	115	371	312	217
AF035020	IGHV3-48	IGHJ3	12 AA	235	0	198	95	183	96
AF035042	IGHV3-53	IGHJ4	12 AA	995	150	102	426	482	664
L12100	IGHV1-2	IGHJ6	12 AA	81	0	0	131	43	159
X54435	IGHV3-74	IGHJ5	12 AA	18	147	0	22	44	89
AF035018	IGHJ2-5	IGHJ4	13 AA	712	354	95	1471	850	792
AF035025	IGHV1-69	IGHJ2	13 AA	0	0	0	39	56	61
AF035041	IGHV4-59	IGHJ4	13 AA	961	248	362	1176	1393	1406
D16837	IGHV1-69	IGHJ4	13 AA	658	206	165	504	815	695
L12061	IGHV1-2	IGHJ4	13 AA	122	80	85	695	631	674
L12102	IGHV1-3	IGHJ4	13 AA	588	360	188	411	440	327
AF035043	IGHV3-43	IGHJ3	14 AA	76	35	77	131	114	126
L12090	IGHV3-53	IGHJ6	14 AA	180	0	113	148	84	116
L12098	IGHV1-3	IGHJ4	14 AA	743	419	138	598	437	449
X73856	IGHV1-8	IGHJ6	14 AA	88	0	0	55	99	160
AF035030	IGHV3-30	IGHJ4	15 AA	2939	1426	828	2052	2534	2339
S73912	IGHV3-21	IGHJ4	15 AA	5	0	0	0	0	0
M85255	IGHV3-30	IGHJ6	16 AA	1878	949	896	1225	1178	1138
U07194	IGHV1-2	IGHJ5	16 AA	50	0	24	163	84	85
AF035022	IGHV1-46	IGHJ4	17 AA	305	123	0	103	284	230
AF035040	IGHV6-1	IGHJ3	17 AA	0	0	162	36	19	0
L12096	IGHV1-69	IGHJ3	17 AA	44	33	23	175	167	164
U07196	IGHV4-61	IGHJ3	17 AA	0	0	0	0	0	0
X73851	IGHV1-2	IGHJ3	17 AA	0	29	5	147	93	126
X73857	IGHV4-31	IGHJ4	17 AA	160	98	204	0	0	0
AF035019	IGHV1-2	IGHJ3	18 AA	0	0	0	60	102	85
AF035027	IGHV3-9	IGHJ1	18 AA	0	0	0	8	24	8
X15611	IGHV1-18	IGHJ4	18 AA	382	154	13	300	277	228
D16833	IGHV3-74	IGHJ2	21 AA	0	0	0	0	0	29
X54445	IGHV4-4	IGHJ4	21 AA	0	0	0	0	0	0
X56592	IGHV4-34	IGHJ4	21 AA	265	95	122	321	170	371
X73859	IGHV3-23	IGHJ6	21 AA	517	200	75	403	583	329
AF035026	IGHV3-15	IGHJ4	22 AA	81	58	5	31	20	10
D84252	IGHV3-33	IGHJ6	22 AA	147	121	0	0	0	0
U07195	IGHV4-34	IGHJ5	22 AA	49	10	0	157	173	100
Total no.				13,921	5986	4386	12,525	13,249	12,929
Percent (%)				2.82	2.35	2.90	2.66	2.52	2.77

## Discussion

BCRs develop with the growth and differentiation of B cells. VDJ rearrangement of BCR heavy chain initially appears in pre-B stage of marrow. After immature B cells leaving marrow and entering into the periphery, some migrating B cells differentiate into immature naïve B cells and further differentiate into mature naïve B cells. The mature naïve B cells go through SHM under antigen stimulation and finally differentiated into memory B cells or plasmocyte secreted antibody. In normal body, this process will not produce autoreactive B cell and autoantibody. However, as for SLE patients, many factors result in the occurrence of autoreactive B cell and autoantibody, including abnormal growth and tolerance of B cell, abnormal activity of B cell and abnormal apoptosis of autoreactive B cell (Renaudineau et al. 2004).

In the past, the technological means to study on BCR repertoire of SLE patients mainly relied on polymerase chain reaction (PCR)-single strand conformation polymorphism analysis (SSCP), flow cytometry and immune spectratyping. Limited by number of sequence data, these past methods were hard to reflect BCR repertoire. High dose glucocorticoid treatment is the most basic treatment plan for SLE patient at the active stage. However, its side effect is very extreme, so many researchers focus on targeted treatment. In the past, it was generally thought that pathopoiesis of SLE was related to T cells and the remission of SLE was related to normal immune reconstitution. However, the current view suggests that autoreactive B cells and autoantibodies are the key factors of pathopoiesis of SLE (Arbuckle et al. 2003); meanwhile, B cells are also considered as the main target of SLE treatment (Shlomchik et al. 2001; Sanz and Lee 2010). Taken together, these reasons prompt us to use high-throughput sequencing to investigate the BCR repertoire of SLE patients after high dose glucocorticoid treatment and screen autoantibody clones.

In this study, we analyzed the composition change of BCR repertoire of two SLE patients after treatment in various angles. In the gene usage, we found that there were some losses of IGHV usages in P1 after treatment, including IGHV3-38, IGHV3-25, IGHV3-7, IGHV3-35 and IGHV3-38-3. As for P2, although there were two new gene expressions with low frequency [IGHV3-7 (0.0025 %) and IGHV3-64D (0.0086 %)] at the first month after treatment, there were still losses of IGHV3-35 and IGHV3-7 gene at the third month after treatment. It was worth noting that the lost two genes of P2 were also in the range of lost gene of P1. Although the frequencies of these lost genes are low, some genes with lower frequency can be detected at each time point. Although these two SLE patients had some losses of gene usages, the number of these genes was not over 100. Such an amount of scale was very small for BCR repertoire. When we investigated the change of IGHV–IGHJ gene pairing over time, these two SLE patients had striking similarity, which no evident change was observed in IGHV–IGHJ pairing at each time point. In addition, analyses of the composition of H-CDR3 showed overall AA compositions of H-CDR3 at three time points in each SLE patient were very similar, and the results of H-CDR3 AA usage that had the same length (14 AA) and the same position were similar. These results support one possibility that this treatment will barely affect the composition of SLE patients’ BCR repertoire in a short term.

Moreover, we have investigated the antinuclear antibodies indexes of these two SLE patients. The expression of some antinuclear antibodies of two SLE patients had decreased after treatment, suggesting the treatment could definitely reduce the formation of autoantibody protein. However, when we screened autoantibody clones, the proportion of potential autoantibody clones of two SLE patients did not shown any decreasing sign. That is, the treatment can reduce the formation of autoantibody in the protein level, but may not reduce the proportion of autoantibody clone in BCR repertoire in the clone level. In the past, we could say that the relief of SLE patients was related to normal immune reconstitution. But now, we must be cautious with this, because our result indicates that the BCR repertoire of these two SLE patients did not turn better. This finding can roughly explain the clinical feature that SLE can easily recur.

In brief, this study has done a short-term analysis and evaluation on BCR repertoire of two SLE patients after high dose glucocorticoid treatment, and this may provide new idea for the immunologic surveillance and treatment of SLE. Moreover, autoantibody sequences that we screened can provide reference for other functional experiments. Finally, we propose that this analysis and evaluation of BCR repertoire can be applied to more other immunologic diseases.

## Conclusion

In this study, our results suggested that high dose glucocorticoid treatment can reduce the formation of autoantibody in the protein level, but may not reduce the proportion of autoantibody clone in BCR repertoire in the clone level. In addition, the treatment in short term may have little impact on compositon of BCR repertoire of SLE patient.

## Additional files

10.1186/s40064-016-1709-4 Clonotype distribution plots of total BCR-H sequences from two SLE patients. Each diamond represents a distinct H-CDR3 amino acid sequence. **A** P1-0 (green), P1-1 (red) and P1-3 (blue). **B** P2-0 (green), P2-1 (red) and P2-3 (blue). **Figure S2.** Length distribution of H-CDR3 AA (position 105-117) in two SLE patients BCR sequences at different time points. Note that the length of the H-CDR3 region according to the definition used by IMGT. **A** P1-0, P1-1 and P1-3. **B** P2-0, P2-1 and P2-3. **Figure S3**. Average values of AA composition calculated for SLE patients VH CDR3 sequences in percent shows the invariability from overall amino acid composition at different time points. The amino acids are arranged by relative hydrophobicity values as found in Kyte-Doolittle scale. **A** P1-0, P1-1 and P1-3; **B** P2-0, P2-1 and P2-3.
10.1186/s40064-016-1709-4 Information of patients. **Table S2.** The clinical hematology parameters of two SLE patients. **Table S3.** Clone no. of IGHV usage with loss of two SLE patients at different time points. **Table S4.** Clonotype (no.) of heavy chain variable regions that use the same V and J genes and have the same CDR3 length as known autoantibody from IMGT/LIGM-DB.
10.1186/s40064-016-1709-4 Putative autoantibody clones.
