# Supplementary material for: Short-term assessment of BCR repertoires of SLE patients after high dose glucocorticoid therapy with high-throughput sequencing
Source: Springerplus. 2016 Jan 26;5:75. doi: 10.1186/s40064-016-1709-4 (PMC4726643; doi:10.1186/s40064-016-1709-4)

**Figure S1** Clonotype distribution plots of total BCR-H sequences from two SLE patients. Each diamond represents a distinct H-CDR3 amino acid sequence. **A** P1-0 (green), P1-1 (red) and P1-3 (blue). **B** P2-0 (green), P2-1 (red) and P2-3 (blue).


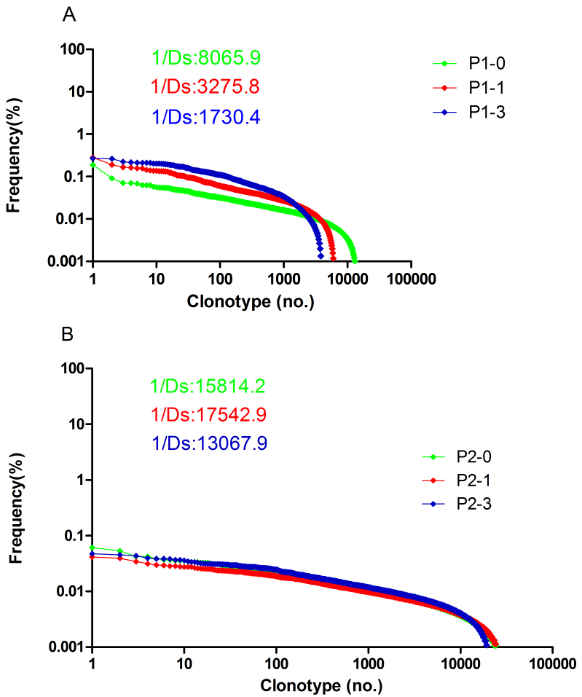


**Figure S2** Length distribution of H-CDR3 AA (position 105-117) in two SLE patients BCR sequences at different time points. Note that the length of the H-CDR3 region according to the definition used by IMGT. **A** P1-0, P1-1 and P1-3. **B** P2-0, P2-1 and P2-3.


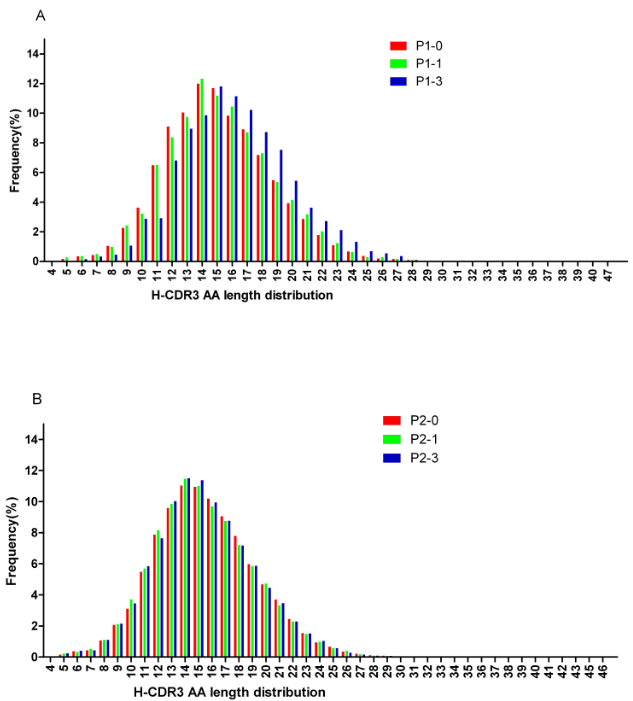


**Figure S3** Average values of AA composition calculated for SLE patients VH CDR3 sequences in percent shows the invariability from overall amino acid composition at different time points. The amino acids are arranged by relative hydrophobicity values as found in Kyte-Doolittle scale (Pommie et al. 2004). **A** P1-0, P1-1 and P1-3; **B** P2-0, P2-1 and P2-3.


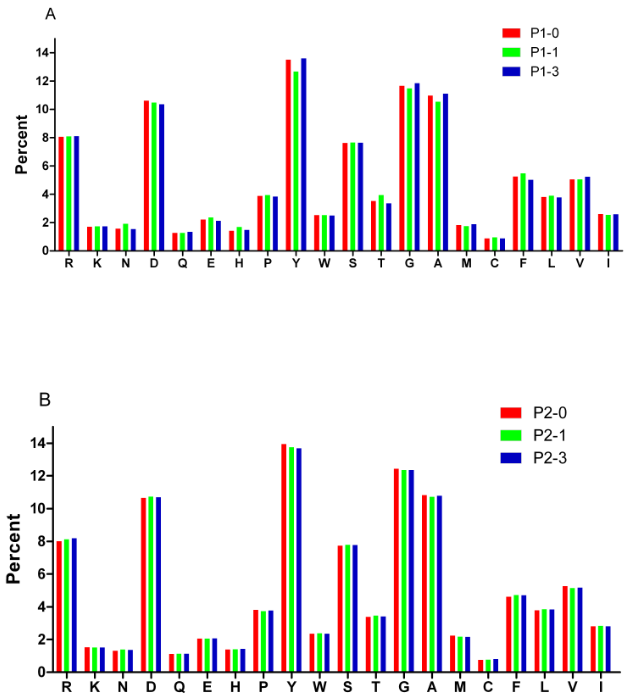

Supplement: Supplementary file 1 — 10.1186/s40064-016-1709-4 Clonotype distribution plots of total BCR-H sequences from two SLE patients. Each diamond represents a distinct H-CDR3 amino acid sequence. A P1-0 (green), P1-1 (red) and P1-3 (blue). B P2-0 (green), P2-1 (red) and P2-3 (blue). Figure S2. Length distribution of H-CDR3 AA (position 105-117) in two SLE patients BCR sequences at different time points. Note that the length of the H-CDR3 region according to the definition used by IMGT. A P1-0, P1-1 and P1-3. B P2-0, P2-1 and P2-3. Figure S3. Average values of AA composition calculated for SLE patients VH CDR3 sequences in percent shows the invariability from overall amino acid composition at different time points. The amino acids are arranged by relative hydrophobicity values as found in Kyte-Doolittle scale. A P1-0, P1-1 and P1-3; B P2-0, P2-1 and P2-3. [file 40064_2016_1709_MOESM1_ESM.docx]
