# Supplementary material for: Short-term assessment of BCR repertoires of SLE patients after high dose glucocorticoid therapy with high-throughput sequencing
Source: Springerplus. 2016 Jan 26;5:75. doi: 10.1186/s40064-016-1709-4 (PMC4726643; doi:10.1186/s40064-016-1709-4)
Supplement: Supplementary file 2 — 10.1186/s40064-016-1709-4 Information of patients. Table S2. The clinical hematology parameters of two SLE patients. Table S3. Clone no. of IGHV usage with loss of two SLE patients at different time points. Table S4. Clonotype (no.) of heavy chain variable regions that use the same V and J genes and have the same CDR3 length as known autoantibody from IMGT/LIGM-DB. [file 40064_2016_1709_MOESM2_ESM.docx]

**Table S1: Information of patients**

| Patients | Gender | Age | Family history | Relapse | Time since SLE diagnosis |
| --- | --- | --- | --- | --- | --- |
| P1 | Female | 45 | No | Yes | 8 years |
| P2 | Male | 20 | No | NO | 1 month |

**Table S2: The clinical hematology parameters of two SLE patients**

| Patients no. | Leucocyte count (×109) | IgM (g/L) | IgA (g/L) | IgG (g/L) | C3 (g/L) |
| --- | --- | --- | --- | --- | --- |
| P1-0 | 7.3 | 0.59 | 2.45 | 7.81 | 0.36 |
| P1-1 | 4.7 | 0.38 | 2.93 | 12.9 | 0.55 |
| P1-3 | 7.1 | 0.37 | 1.82 | 13.7 | 0.42 |
| P2-0 | 6.8 | 0.67 | 1.73 | 14.92 | 0.30 |
| P2-1 | 3.6 | 0.27 | 0.60 | 5.32 | 0.46 |
| P2-3 | 7.5 | 0.20 | 0.57 | 3.18 | 0.81 |

Reference value：Leucocyte 4-10, IgM 0.46-3.04, IgA 0.82-4.53, IgG 7.51-15.6, C3 0.79-1.52

**Table S3: Clone no. of IGHV usage with loss of two SLE patients at different time points**

| IGHV genes | P1-0 | P1-1 | P1-3 | P2-0 | P2-1 | P2-3 |
| --- | --- | --- | --- | --- | --- | --- |
| IGHV3-38 | 9 | 0 | 0 | 0 | 0 | 0 |
| IGHV3-25 | 9 | 0 | 0 | 0 | 0 | 0 |
| IGHV3-7 | 57 | 0 | 0 | 0 | 13 | 0 |
| IGHV3-35 | 27 | 30 | 0 | 13 | 30 | 0 |
| IGHV3-38-3 | 99 | 100 | 0 | 4 | 71 | 0 |

**Table S4: Clonotype (no.) of heavy chain variable regions that use the same V and J genes and have** **the same CDR3 length as known autoantibody from IMGT/LIGM-DB**

| Autoantibody accession no. | P1-0 | P1-1 | P1-3 | P2-0 | P2-1 | P2-3 |
| --- | --- | --- | --- | --- | --- | --- |
| AF035024 | 2 | 0 | 0 | 3 | 7 | 4 |
| AF035021 | 28 | 9 | 8 | 51 | 53 | 68 |
| L12105 | 1 | 1 | 1 | 11 | 19 | 9 |
| AF035023 | 3 | 2 | 2 | 6 | 9 | 3 |
| L12087 | 14 | 8 | 4 | 17 | 20 | 10 |
| AF035020 | 5 | 0 | 3 | 10 | 11 | 8 |
| AF035042 | 23 | 4 | 4 | 28 | 33 | 33 |
| L12100 | 2 | 0 | 0 | 5 | 3 | 4 |
| X54435 | 1 | 4 | 0 | 3 | 2 | 5 |
| AF035018 | 18 | 7 | 3 | 67 | 43 | 32 |
| AF035025 | 0 | 0 | 0 | 2 | 3 | 1 |
| AF035041 | 32 | 8 | 9 | 82 | 88 | 72 |
| D16837 | 28 | 8 | 6 | 43 | 58 | 41 |
| L12061 | 6 | 2 | 3 | 48 | 45 | 40 |
| L12102 | 19 | 9 | 7 | 24 | 32 | 18 |
| AF035043 | 1 | 1 | 1 | 9 | 5 | 6 |
| L12090 | 2 | 0 | 2 | 8 | 3 | 4 |
| L12098 | 23 | 12 | 6 | 32 | 31 | 21 |
| X73856 | 2 | 0 | 0 | 1 | 3 | 3 |
| AF035030 | 107 | 52 | 34 | 188 | 195 | 141 |
| S73912 | 1 | 0 | 0 | 0 | 0 | 0 |
| M85255 | 37 | 17 | 18 | 57 | 53 | 36 |
| U07194 | 2 | 0 | 1 | 12 | 10 | 8 |
| AF035022 | 14 | 3 | 0 | 12 | 23 | 16 |
| AF035040 | 0 | 0 | 2 | 3 | 1 | 0 |
| L12096 | 3 | 3 | 1 | 18 | 14 | 14 |
| U07196 | 0 | 0 | 0 | 0 | 0 | 0 |
| X73851 | 0 | 2 | 1 | 9 | 10 | 8 |
| X73857 | 3 | 3 | 2 | 0 | 0 | 0 |
| AF035019 | 0 | 0 | 0 | 7 | 11 | 3 |
| AF035027 | 0 | 0 | 0 | 1 | 3 | 3 |
| X15611 | 17 | 8 | 2 | 35 | 28 | 19 |
| D16833 | 0 | 0 | 0 | 0 | 0 | 1 |
| X54445 | 0 | 0 | 0 | 0 | 0 | 0 |
| X56592 | 7 | 3 | 1 | 18 | 7 | 17 |
| X73859 | 13 | 4 | 1 | 23 | 28 | 12 |
| AF035026 | 3 | 2 | 1 | 6 | 2 | 1 |
| D84252 | 2 | 2 | 0 | 0 | 0 | 0 |
| U07195 | 2 | 1 | 0 | 9 | 8 | 8 |
| Total no. | 421 | 175 | 123 | 848 | 861 | 669 |
| Clone no.≥50 | 85 | 45 | 34 | 8 | 5 | 14 |
